# High-Temperature Oxidation of NbTi-Bearing Refractory Medium- and High-Entropy Alloys

**DOI:** 10.3390/ma17184579

**Published:** 2024-09-18

**Authors:** Wei-Chih Lin, Yi-Wen Lien, Louis Etienne Moreau, Hideyuki Murakami, Kai-Chi Lo, Stéphane Gorsse, An-Chou Yeh

**Affiliations:** 1 Department of Materials Science and Engineering, National Tsing Hua University, 101, Section 2, Kuang-Fu Road, Hsinchu 300044, Taiwan; 2 Centre National de la Recherche Scientifique (CNRS), University Bordeaux, Bordeaux INP, ICMCB, UMR 5026, F-33600 Pessac, France; 3 High Entropy Materials Center, National Tsing Hua University, 101, Section 2, Kuang-Fu Road, Hsinchu 300044, Taiwan; 4 Research Center for Structural Materials, National Institute for Materials Science, 1-2-1 Sengen, Tsukuba 305-0047, Japan; 5 Department of Nanoscience and Nanoengineering, Waseda University, 3-4-1 Okubo, Shinjuku, Tokyo 169-8555, Japan

**Keywords:** refractory high-entropy alloys, chemical segregation, oxidation

## Abstract

The oxidation of six NbTi-i refractory medium- and high-entropy alloys (NbTi + Ta, NbTi + CrTa, NbTi + AlTa, NbTi + AlMo, NbTi + AlMoTa and NbTi + AlCrMo) were investigated at 1000 °C for 20 h. According to our observation, increased Cr content promoted the formation of protective CrNbO_4_ and Cr_2_O_3_ oxides in NbTi + CrTa and NbTi + AlCrMo, enhancing oxidation resistance. The addition of Al resulted in the formation of AlTi-rich oxide in NbTi + AlTa. Ta addition resulted in the formation of complex oxides (MoTiTa_8_O_25_ and TiTaO_4_) and decreased oxidation resistance. Meanwhile, Mo’s low oxygen solubility could be beneficial for oxidation resistance while protective Cr_2_O_3_/CrNbO_4_ layers were formed. In NbTi + Ta, NbTi + AlTa and NbTi + CrTa, a considerable quantity of Ti-rich oxide was observed at the interdendritic region. In NbTi + AlCrMo, the enrichment of Cr and Ti at the interdendritic region could fasten the rate of oxidation. Compared to the recent research, NbTi + AlCrMo alloy is a light-weight oxidation-resistant alloy (weight gain of 1.29 mg/cm^2^ at 1000 °C for 20 h and low density (7.2 g/cm^3^)).

## 1. Introduction

Global warming has become a critical issue in recent years due to the extensive usage of fossil fuels, mainly in power plants and engines [1]. Therefore, the engine’s efficiency needs to be improved to reduce the emission of CO_2_. Better performance and efficiency could be acquired at higher operation temperatures [2]. Ni-base superalloys have been critical materials since the last century due to their excellent high-temperature properties. However, the operating temperature of newly invented turbine engines is over the melting point of Ni and relies on the cooling channel design [3] and thermal barrier coating [4]. If greater efficiency is required, new materials with greater temperature capability need to be developed.

Refractory high-entropy alloys (RHEAs) have been considered as potential candidates for high-temperature applications, such as aerospace engines, gas turbines and nuclear reactors. They were first reported by O.N. Senkov et al. [5]. These alloys are composed of high-melting-temperature elements, which are usually higher than Ni, under the concept of high-entropy alloys, in which the atomic percentage of each element varies from 5% to 35% with more than five elements. The most well-known advantages of RHEAs are their high-temperature mechanical properties [5,6,7,8,9]. However, it is important to note that the development of these alloys is still limited by their poor oxidation resistance [10,11,12]. In order to design RHEAs with improved oxidation resistance, it is important to consider the effects of each element on oxidation properties. Recent studies [13,14,15,16] have demonstrated the potential of oxidation resistance in RHEAs. In Lo’s work [13,14], Cr-17.6Al-20.3Mo-15.2Nb-2.9Si-13.4Ta-5.4Ti with CrTaO_4_ oxide formation showed only 4 mg/cm^2^ mass gain at 1100 and 1200 °C for 200 h oxidation. Several studies [17,18,19,20,21,22,23,24,25,26] have elucidated the relationship between some elemental effects and oxidation in RHEAs. In Müller et al. research [25], AlCrMoTaTi, AlCrMoNbTi, AlCrMoTa and AlCrMoNb were fabricated to study the effect of Nb, Ta and Ti. Rutile-type oxides, such as CrNbO_4_ and CrTaO_4_, demonstrated protective properties during oxidation. The addition of Ti was found to reduce the formation of Nb_2_O_5_ and Ta_2_O_5_ and facilitate the formation of rutile-type oxides. AlCrNbTaTiZr was studied to show that Al can reduce the inward diffusion of oxygen, and Zr can suppress the cracking in the oxide scale [18].

To further understand the elemental effects on oxidation in RMEAs and RHEAs, in this work, we fabricated and tested six refractory alloy compositions: one ternary (NbTi + Ta), three quaternary (NbTi + CrTa, NbTi + AlTa, NbTi + AlMo) and two senary (NbTi + AlMoTa, NbTi + AlCrMo). This comprehensive investigation aims to elucidate the interplay between these factors and guide the development of RHEAs with tailored oxidation resistance properties for future applications.

## 2. Materials and Methods

### 2.1. Material Design

**Figure 1:**
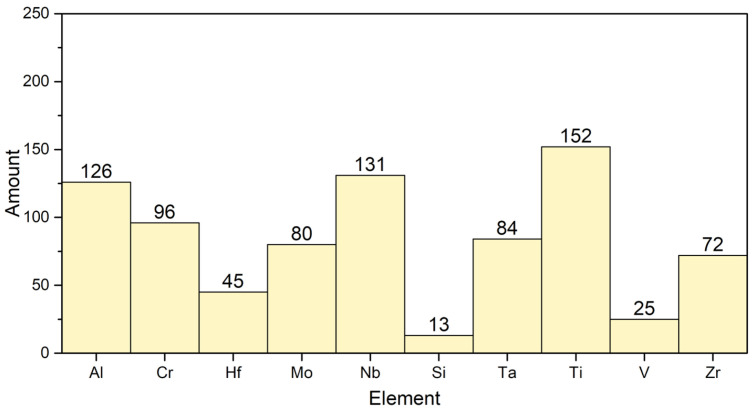
The statistics of element presence in RHEAs from the literature collection [7,13,14,15,16,17,19,20,21,22,25,27,28,29,30,31,32,33,34,35,36,37,38,39,40,41,42,43,44,45,46,47,48,49,50,51,52,53,54,55].

In this work, NbTi-bearing refractory alloys with different compositions were designed. The choice of elements was based on the most common occurrence in the oxidation research of RHEAs in recent years [7,13,14,15,16,17,19,20,21,22,25,27,28,29,30,31,32,33,34,35,36,37,38,39,40,41,42,43,44,45,46,47,48,49,50,51,52,53,54,55] (Figure 1).

To avoid the high-fraction intermetallic phase that induced brittleness and caused fabrication difficulties, alloys were designed with mainly a single BCC phase. The designation of these six alloys are NbTi + Ta, NbTi + CrTa, NbTi + AlTa, NbTi + AlMo, NbTi + AlCrMo and NbTi + AlMoTa, and the actual compositions are presented in the next section.

### 2.2. Experiment Procedure

The ingots were prepared by vacuum arc melting high-purity materials (>99.99 wt%) on a water-cooled copper mold under the pure argon atmosphere. The button-shaped ingots were flipped and re-melted three times to ensure chemical homogeneity. The compositions of the as-cast samples are shown in Table 1.

All samples were cut by electric discharging machining. The as-cast samples for scanning electron microscope (SEM) observation were polished with 0.05 μm Al_2_O_3_ dispersion fluid. The Zeiss Gemini 300 SEM (Zeiss, Oberkochen, Germany) equipped with EDS was utilized to observe the microstructures and chemical compositions of the phases. The X-ray diffraction (XRD) patterns of the as-cast samples were collected with a Bruker D2 Phaser (Bruker, Billerica, MA, USA) equipped with a Cu target. Each measurement was made within an angular range of 2θ = 20–100° with a scanning rate of 5.7° per min. The SearchMatch software 2.1.1.1. with the Powder Diffraction File 2 database was utilized to characterize the XRD result. Thermo-Calc 2024b, a thermodynamic software program utilizing the TCHEA4 database, was employed to elucidate the behavior of phase formation and element segregation.

The samples for thermogravimetric analysis (TGA) were 2.5 × 2.5 × 5.5 mm in dimensions and ground with 1200 grits SiC abrasive paper for all surfaces. The oxidation tests were conducted at 1000 °C for 20 h in laboratory air with a thermogravimetric analyzer (TGA)-SETARAM TAG 24-18S (SETARAM, Caluire, France) simultaneous symmetrical thermoanalyzer. To further examine the microstructure during the oxidation test, isothermal oxidations were performed in a box furnace at 1000 °C for 20 h.

The XRD patterns of oxidized samples were collected with a PANalytical X’pert PRO MPD (Malvern Panalytical, Almelo, The Netherlands) diffractometer in Bragg–Brentano θ–2θ geometry equipped with a secondary monochromator, an X’Celerator multi-strip detector and a 3 × 15 sample exchanger. Each measurement was made within an angular range of 2θ = 8–100° with a scanning rate of 2.1° per min. The Cu-Kα radiation was generated at 45 kV and 40 mA (λ = 0.15418 nm). The samples with clay below were put on sample holders made of aluminum alloy and flattened with a piece of glass. The SearchMatch software with the Powder Diffraction File 2 database was utilized to characterize the XRD result. The oxidized samples for SEM observation were mounted in the epoxy resin and polished with 0.05 μm Al_2_O_3_ dispersion fluid. The oxidized microstructure was observed with a Zeiss Gemini 300 SEM equipped with EDS.

## 3. Results

### 3.1. As-Cast Microstructure and Segregation Analysis

**Figure 2:**
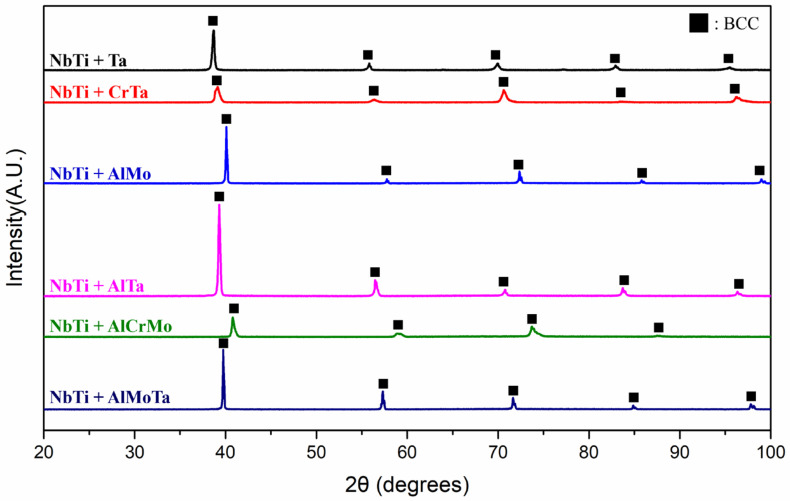
The XRD patterns of NbTi-bearing RMEAs and RHEAs.

The XRD analysis of the crystal structure revealed that all fabricated alloys exhibited the BCC phase (Figure 2).

**Figure 3:**
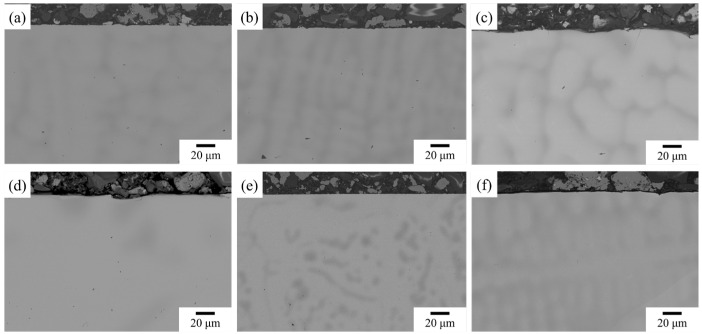
The as-cast microstructure of (**a**) NbTi + Ta, (**b**) NbTi + CrTa, (**c**) NbTi + AlMo, (**d**) NbTi + AlTa, (**e**) NbTi + AlCrMo and (**f**) NbTi + AlMoTa near the surface area.

The images (Figure 3) showed that the materials exhibited the typical dendrite and interdendritic structure.

Table 2 provides a summary of the chemical analysis results indicated in the BEI. Al, Cr and Ti are strongly segregated at the interdendritic region, while Mo and Ta are distributed on the dendrites. Nb shows no obvious segregation in NbTi + Ta and NbTi + AlMoTa, but it shows segregation in NbTi + AlTa, NbTi + AlMo, NbTi + CrTa and NbTi + AlCrMo.

### 3.2. TGA and Oxidized Microstructure

**Figure 4:**
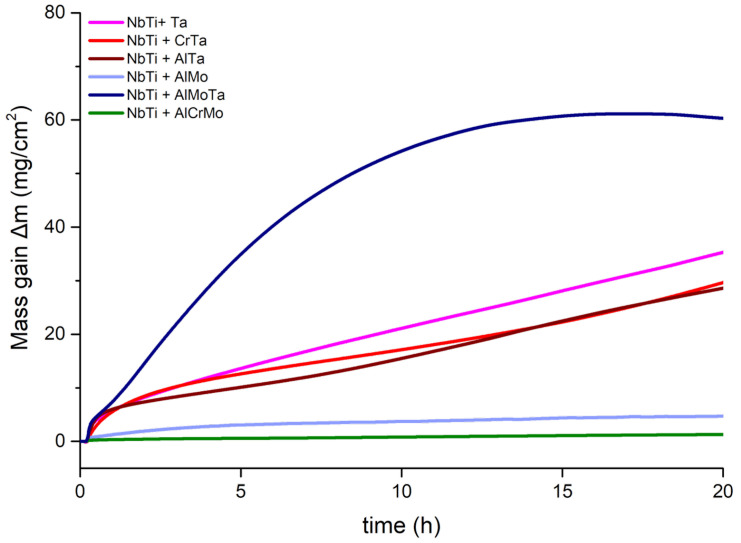
The oxidation mass gain curve tested at 1000 °C for 20 h.

Figure 4 illustrates the mass gain curves of NbTi-bearing RMEAs and RHEAs at 1000 °C up to 20 h.

To further understand the oxidation mass gain behavior of the investigated alloys, the following equation was used:

(1) $$∆m=kt^{n}$$

where ∆m is the mass gain of each alloy, *k* is the oxidation rate constant, and *n* is the time exponent. In this research, most of the alloys exhibited two-stage oxidation behaviors (Table 3), except NbTi + AlMoTa. For the NbTi + AlCrMo, the oxidation behavior was found to obey cubic growth kinetics in the first stage. After 7 h of oxidation, the time exponent (*n*) increased to 0.59, indicating that the oxidation kinetics were parabolic. The time exponent in the first stage is 0.55, 0.46 and 0.34 for NbTi + Ta, NbTi + CrTa and NbTi + AlTa, respectively. Due to the higher oxidation rate constant (*k*), the mass gain of these alloys is considerably greater than NbTi + AlCrMo. In the second stage, the time exponent of NbTi + Ta, NbTi + CrTa and NbTi + AlTa increased to 0.89, 1.12 and 0.84, respectively. The oxidation kinetics of NbTi + Ta, NbTi + CrTa and NbTi + AlTa obey the linear growth rate for the second stage. The time exponent for NbTi + AlMo was higher in the first stage than the alloys mentioned above. For NbTi + AlMoTa, the oxidation rate constant was the largest in the first stage.

**Figure 5:**
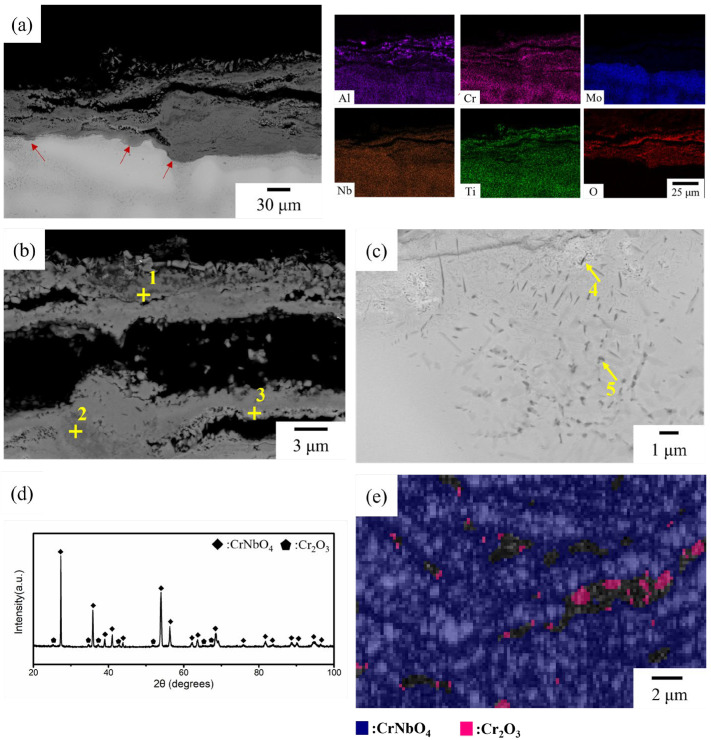
The oxidized NbTi + AlCrMo for 20 h at 1000 °C: (**a**) a general view of the oxide layer with EDS mapping, the magnified SEM image of (**b**) the external oxidation region and (**c**) the internal oxidation region and (**d**) the XRD pattern of the oxide layer. (**e**) The EBSD image with the band contrast and phase characterization of the external oxide region. The red arrows in (**a**) indicates the oxidation of the interdendritic region. The compositions of the indicated regions in (**b**,**c**) are listed in Table 4.

NbTi + AlCrMo exhibited exceptional oxidation resistance in this work, as it was the only alloy to form an external oxide layer (Figure 5a).

It can be observed that the alloy element diffuses outward and combines with oxygen to form oxides. Microstructure characterization revealed a laminar structure consisting of the Al-Cr-depleted region and Al-rich and Cr-rich oxide layer. The interdendritic area, which contains higher levels of Al, Cr and Ti, was observed to oxidize at a faster rate than the dendritic area located at the border between the external and internal oxide areas. The external oxide layer is composed of at least three types of oxides (Table 4), as characterized by EDS: Al-rich, Cr-rich and Nb-rich. Within the internal oxidation region, two types of particles were identified. The first was needle-shaped and rich in Ti and O, while the second was cuboidal and rich in Ti and N. XRD analysis revealed that the external oxide layer is composed of Cr_2_O_3_ and CrNbO_4_ with a rutile structure (Figure 5d). This type of oxide, which has a continuous morphology, can effectively prevent inward oxygen diffusion. However, it should be noted that at high temperatures, the evaporation of Mo oxide led to the creation of significant porosities in the oxide layer. The EBSD result also showed the main oxides in the external oxidation region are Cr_2_O_3_ and CrNbO_4_, which have 1.8% and 98.2%, respectively (Figure 5e).

**Figure 6:**
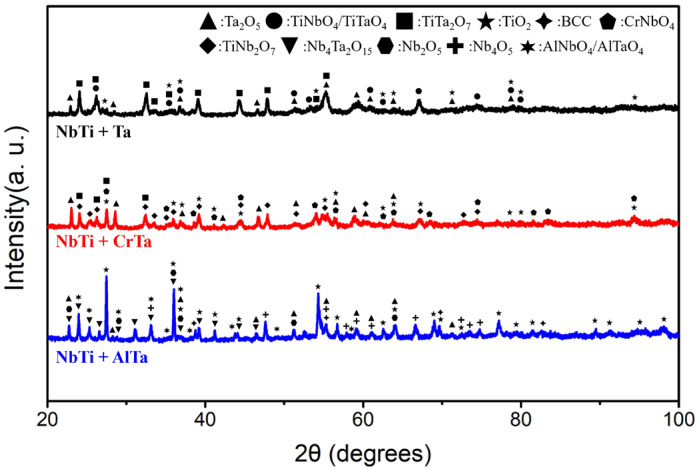
The XRD patterns of NbTi + Ta, NbTi + CrTa and NbTi + AlTa after oxidation at 1000 °C for 20 h.

Comparing the XRD results of NbTi + Ta, NbTi + CrTa and NbTi + AlTa, TiO_2_ and Ta_2_O_5_ are the common products among them (Figure 6).

Besides the simple oxides, several complex oxides were also characterized, such as TiTa_2_O_7_ in NbTi + Ta and NbTi + CrTa and Nb_4_Ta_2_O_15_ in NbTi + AlTa. In addition, NbTi + CrTa yielded CrNbO_4_ after 20 h of oxidation. In NbTi + AlTa, there is AlNbO_4_ oxide characterized by XRD.

**Figure 7:**
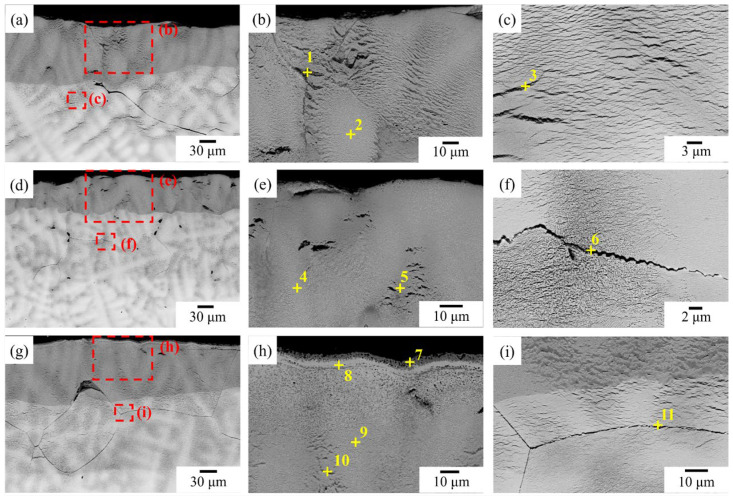
The BEI images after oxidation at 1000 °C for 20 h: a general view of the oxide layer of (**a**) NbTi + Ta, (**d**) NbTi + CrTa and (**g**) NbTi + AlTa, the magnified SEM image of the external oxidation region of (**b**) NbTi + Ta, (**e**) NbTi + CrTa and (**h**) NbTi + AlTa and the internal oxidation region of (**c**) NbTi + Ta, (**f**) NbTi + CrTa and (**i**) NbTi + AlTa. The compositions of the indicated regions in (**b**,**c**,**e**–**h**) are listed in Table 5.

A cross-sectional SEM-EDS analysis of NbTi + Ta, NbTi + CrTa and NbTi + AlTa revealed comparable oxidized microstructures following 1000 °C oxidation for 20 h (Figure 7).

The formation mechanism of the oxide layer differs from that observed in NbTi + AlCrMo. In NbTi + Ta, NbTi + CrTa and NbTi + AlTa, the inward diffusion of oxygen atoms is a significant factor in oxide formation. The oxide layer exhibits a dendrite structure similar to the metal part. A significant quantity of Ti-rich oxide was observed in the interdendritic region and inner oxide layers (Regions 1, 3, 5, 6,10 and Table 5). Additionally, NbTa-rich oxide was identified in the dendrite region (Regions 2, 4 and 9). Furthermore, two thin oxide layers, which are rich in Al-Ti and Nb-Ta, respectively, were observed in NbTi + AlTa (Regions 7 and 8). Their microstructures are distinct from the oxide layer below. The thickness of the first oxide layer was found to be 51.2, 56.0 and 81.4 μm for NbTi + Ta, NbTi + CrTa and NbTi + AlTa, respectively. A significant quantity of Ti-rich oxide was observed in the grain boundary and interdendritic region of the inner oxidation layer.

**Figure 8:**
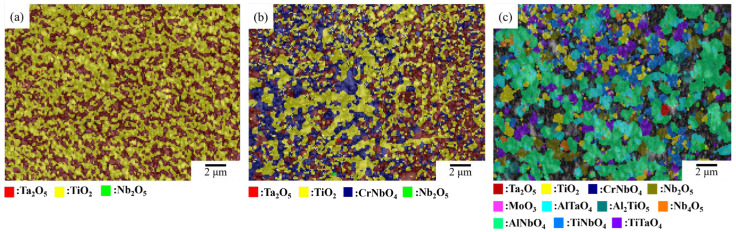
The EBSD band contrast and phase characterization images of (**a**) NbTi + Ta, (**b**) NbTi + CrTa and (**c**) NbTi + AlTa.

The EBSD characterization (Figure 8 and Table 6) shows a large fraction of TiO_2_ and Ta_2_O_5_ in NbTi + Ta and NbTi + CrTa. In addition, CrNbO_4_ dispersed in the oxide layer of NbTi + CrTa. However, in contrast to the SEM observation results, CrNbO_4_ did not form a continuous layer that could protect the alloy. In NbTi + AlTa, the main oxides were AlNbO_4_, TiNbO_4_, TiTaO_4_ and AlTaO_4_. As with the SEM observation, the oxides also showed an uneven distribution. Al-free oxides accumulated in a specific region, which may have been the original dendrite zone.

**Figure 9:**
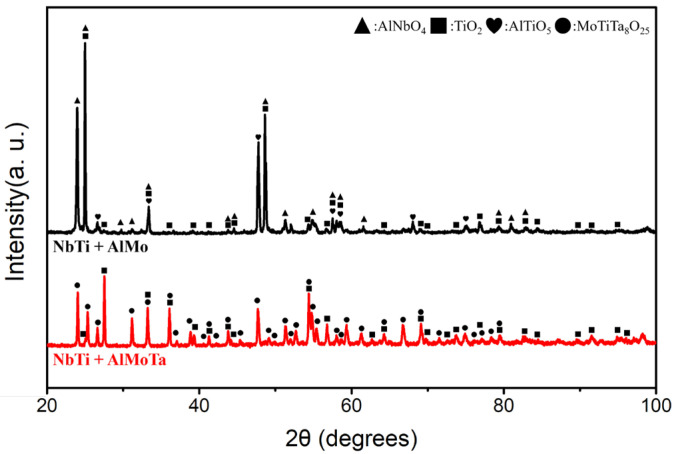
The XRD patterns of NbTi + AlMo and NbTi + AlMoTa after oxidation at 1000 °C for 20 h.

The XRD characterization result indicated that TiO_2_ was the common oxide in NbTi + AlMo and NbTi + AlMoTa (Figure 9).

In addition to TiO_2_, AlNbO_4_ and Al_2_TiO_5_ were yielded in NbTi + AlMo after 1000 °C oxidation for 20 h. In NbTi + AlMoTa, MoTiTa_8_O_25_ was identified as another product after oxidation.

**Figure 10:**
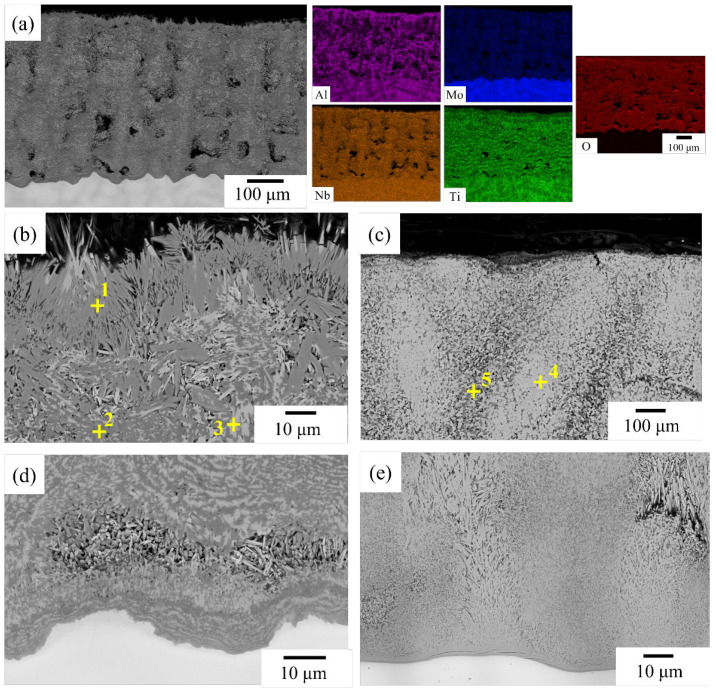
(**a**) The oxide layer BEI with EDS mapping and (**b**) the magnified image of NbTi + AlMo after 1000 °C oxidation for 20 h. (**c**) The oxide layer BEI of NbTi + AlMoTa after 1000 °C oxidation for 20 h. The BEI of the interface between the oxide layer and oxygen-affected zone for (**d**) NbTi + AlMo and (**e**) NbTi + AlMoTa after 1000 °C oxidation for 20 h. The compositions of the indicated regions in (**b**,**c**) are listed in Table 7.

In a cross-sectional SEM image, there is no external oxide layer in NbTi + AlMo and NbTi + AlMoTa. Furthermore, numerous porosities were observed in the oxide layer of NbTi + AlMo following 20 h of oxidation. The thickness of the oxide layer was approximately 471 μm and 2.8 mm in NbTi + AlMo and NbTi + AlMoTa, respectively (Figure 10a).

The EDS mapping of NbTi + AlMo revealed the distribution of elements in the oxide layer. In comparison to the other alloy elements, the content of Mo was found to be significantly lower, and an Al-Nb-rich oxide layer was observed to have formed at the surface of NbTi + AlMo. In the oxide layer, the presence of AlNbO_4_ was identified at the surface of the oxide layer in NbTi + AlMo (Figure 10b and Table 7).

Inside the oxide layer, the Nb-rich oxide and Ti-rich oxide were identified at the original dendrite and interdendritic region. In NbTi + AlMoTa, the presence of Ta oxide in the original dendrite region and Ti-rich oxide in the interdendritic region was observed in the oxide layer (Figure 10c). At the interface between the oxide layer and the oxygen-affected zone, no Ti-rich oxide was observed in the interdendritic region of these alloys (Figure 10d,e). The internal oxide region is hard to observe in both alloys.

**Figure 11:**
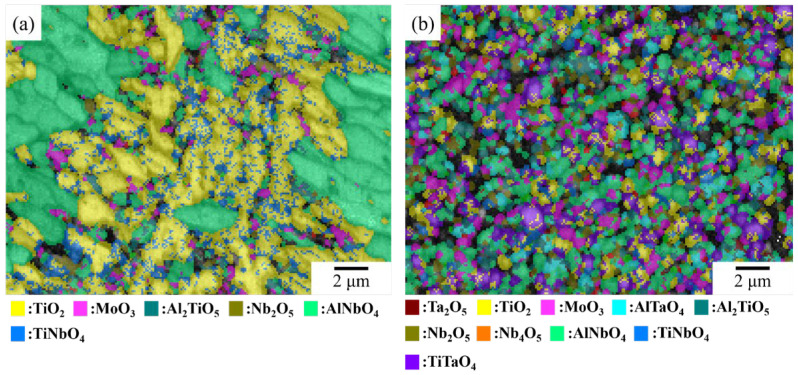
The EBSD band contrast and phase characterization images of (**a**) NbTi + AlMo and (**b**) NbTi + AlMoTa.

In the EBSD observation, the layer structure in NbTi + AlMo is mainly composed of TiO_2_ and AlNbO_4_ (Figure 11a and Table 8). Meanwhile, TiNbO_4_, Al_2_TiO_5_ and MoO_3_ dispersed in the TiO_2_ layer. In NbTi + AlMoTa, several complex oxides with MoO_3_ and TiO_2_ were found to be dispersed within the oxide layer, along with the presence of large amounts of porosity. This result is in accordance with the SEM and XRD characterization.

## 4. Discussion

### 4.1. Elemental Segregation

According to Figure 3 and Figure 7, the samples exhibited visible dendritic microstructure, which indicates a certain degree of chemical segregation. In order to investigate the element distributions, 20 × 20 point metrics were measured, and the composition results were fitted with the Scheil equation, which is shown in the following equation [56].

(2) $$C_{s}=k\;C_{0}\;{\left(1-f_{s}\right)}^{k-1}$$

where $C_{s}$ is the element concentration, *k* is the partition coefficient, $C_{0}$ is the mean composition, and $f_{s}$ is the volume fraction of the solid. The calculation result is concluded in Table 9.

The element tends to segregate in the dendritic region with a value larger than 1 and vice versa. When the value is equal to 1, the element has the same tendency to distribute in the dendritic and interdendritic regions. Al and Cr tend to segregate in the interdendritic region. The segregation of Mo and Nb in the dendritic region is much stronger in NbTi + AlCrMo. In an alloy containing Al, Cr and Ti, Al and Cr are the main elements that segregate into the interdendritic region as reported in the previous work [55].

It can be identified that both Mo and Ta have a preference for distribution in the dendritic region. A comparison of the NbTi + AlMo, NbTi + AlTa and NbTi + AlMoTa samples reveals that the addition of Mo and Ta has the effect of inducing further segregation of Al and Ti to the interdendritic region. According to the binary phase diagram, it can be observed that Mo, Nb and Ta are fully soluble in each other [57]. However, their solubility toward Al is relatively low [58,59,60]. The mixing enthalpy of the Ti with Nb, Mo and Ta is higher, as shown in Table 10, indicating a repulsive interaction between the elements [61,62].

From this perspective, it can also explain the stronger segregation of Mo in NbTi + AlCrMo compared to NbTi + AlMo. The high mixing enthalpy pushes Cr and Mo into the interdendritic and dendritic regions, respectively.

The activities of alloying elements related to the formation of the oxide have been reported in some research [64,65]. In this work, the activities of each alloying element shown in Table 11 were obtained from Thermo-calc calculation.

Ti exhibits significantly higher activity than other elements within NbTi-bearing RMAS and RHEAs. This elevated activity likely explains the substantial formation of Ti-rich oxides, particularly within the interdendritic regions of NbTi + Ta, NbTi + CrTa and NbTi + AlTa alloys. In the interdendritic region, a considerable quantity of Ti-rich oxide was distributed in the oxygen-affected zone. Ti-rich oxide was also distributed in the dendritic region in the lower-partition-degree alloy, such as NbTi + AlMo. However, this phenomenon is absent in NbTi + AlMoTa. It showed a stronger partition effect, which means Ti has a higher content in the interdendritic region, while Nb and Ta are more prevalent in the dendritic region. During oxidation, several oxides can typically react and form complex oxides [15,23,25,31]. However, the uneven distribution results in a greater yield of Ti-rich oxide in the interdendritic region. TiO_2_ also has lower formation free energy than Nb_2_O_5_ and Ta_2_O_5_ [24]. In addition to the interdendritic region, grain boundaries are another location where Ti-rich oxide is distributed. The oxygen has a faster diffusion rate at grain boundaries. Grain boundaries also provide nucleation sites for oxides. Consequently, TiO_2_ nucleates at the grain boundaries, and the width of TiO_2_ is thicker when grain boundaries travel across the interdendritic region.

Cr is another element displaying a high activity. Comparing the oxide layers formed on NbTi + CrTa and NbTi + AlCrMo alloys, NbTi + AlCrMo managed to form a protective layer, while NbTi + CrTa oxidized internally with the original dendritic structure and lacks protective qualities. This disparity might be attributed to the differing activities and diffusivities of Nb and Cr. The diffusivities of Cr and Nb in NbTi + CrTa are 4.48 × 10^−14^ and 1.34 × 10^−15^ m^2^/s, respectively. In NbTi + AlCrMo, the diffusivities of Cr and Nb are 4.23 × 10^−14^ and 3.07 × 10^−18^ m^2^/s, respectively. In NbTi + CrTa, Nb’s high diffusivity and activity lead to an increased formation rate of Nb_2_O_5_, which possesses a polymorphic structure [24,25,66,67]. This polymorphic phenomenon can induce volume changes in the oxide layer and result in crack formation. Although Cr_2_O_3_ can combine with Nb_2_O_5_ to form CrNbO_4_ [28], the limited amount of Cr is insufficient to entirely prevent Nb_2_O_5_ formation. Conversely, the low diffusivity of Nb in NbTi + AlCrMo restricts its outward diffusion, thereby reducing the formation rate of Nb_2_O_5_.

### 4.2. The Oxidation Mechanism of NbTi-Bearing Refractory High Entropy

#### 4.2.1. Al-Contained NbTi-Bearing RMEAs and RHEAs

In NbTi + AlTa, NbTi + AlMo and NbTi + AlCrMo, the Al-included complex oxide layers were observed at the surface. NbTi + AlCrMo is the only alloy that formed external oxide layers with protective properties. When comparing the oxides formed in the RHEAs, it was found that Al_2_O_3_ and TiO_2_ have lower standard free energy per mole oxygen [25]. Al and Ti diffused outward and reacted with oxygen at the beginning of oxidation. In NbTi + AlTa, the complex (Al, Ti) oxide layer formed also brought a depletion region, which was enriched in Nb and Ta after Al and Ti had diffused outward. This oxide layer is unable to block the oxygen diffused inward. After the NbTa-rich layer was oxidized, the diffusion of Al and Ti was limited, and the growth of the complex (Al, Ti) oxide layer was stopped. Although the continuous Al_2_O_3_ oxide layer can block oxygen and nitrogen diffusion inward to the material during oxidation [68], the formation of the Al_2_O_3_ is rarely seen in RHEAs, even with the high amount of Al addition [21,37,55]. In the interdendritic region of NbTi + AlTa, the second layer of the complex (Al, Ti) oxide is observed (Figure 7h). At the interface of the NbTa-rich oxide layer and alloy, the inward-diffused oxygen reacted again with Al and Ti. However, this oxide formation cycle did not proceed in the same manner as the previous cycle. The inward diffusion of O was much faster than the growth rate of the AlTi complex oxide layer, resulting in a significantly reduced thickness of the AlTi complex oxide layer compared to the first external oxide layer.

In contrast to other Al-included RHEAs, NbTi + AlMoTa did not form such Al-rich oxide layers at the surface. As shown in Table 7, among all Al-included RMEAs and RHEAs, NbTi + AlMoTa has the highest partitioning ratio of Al. The greater inhomogeneity of Al makes it more challenging for the alloy to form a homogeneous Al-oxide layer.

The external oxide layer in NbTi + AlCrMo is a crucial factor in better oxidation resistance. The formation of the external oxide layer in NbTi + AlCrMo occurs through three steps. The first step is Al and Ti outward diffusion and reaction. Once the Al and Ti oxides have diffused outward and reacted with oxygen, the Cr and Nb oxides become the lowest standard free energy in the depletion region [25]. Previous research [13,55] has demonstrated that the principal oxides in the Cr-rich oxide layer, namely CrNbO_4_ and Cr_2_O_3_, exhibit good oxidation resistance. The continuous Cr-oxide layer impedes inward oxygen diffusion. In the final stage, Mo is the sole element present in the depletion region. The EDS result (Figure 10a) indicates that the Mo content at the interface between the external oxide layer and the material is higher than in the inner region. During oxidation, Mo reacts with oxygen to form MoO_3_. This oxide evaporates at temperatures above 800 °C [69]. The evaporation of Mo resulted in the formation of numerous cracks or voids in the vicinity of the Cr-rich oxide layer. Subsequent to the oxidation of the Mo-rich region and the evaporation of Mo oxide, the oxidation process reverted to the initial stage.

#### 4.2.2. The Oxidation Mechanism Difference between NbTi + Ta, NbTi + AlTa and NbTi + CrTa

Comparing NbTi + Ta, NbTi + AlTa and NbTi + CrTa, NbTi + Ta exhibits the highest mass gain during oxidation. Following the addition of Cr, NbTi + CrTa begins to form CrNbO_4_. However, the limited amount of Cr is insufficient to create a continuous oxide layer independently. The improvement is not discernible. The more pronounced segregation effect of Cr can be observed with the lower amount of Cr [70]. In this context, Cr oxide is unable to form homogeneously and react with other oxides to form complex oxides with protective properties. The oxidation properties are marginally enhanced in NbTi + AlTa in comparison to NbTi + Ta. Al continues to benefit from the oxidation properties with the low amount of Al addition. However, in previous literature, Cr provided superior oxidation resistance than Al under the equimolar RHEAs [55]. In this study, the oxidation behavior of NbTi + CrTa and NbTi + AlTa was compared. Al addition was found to have a lower segregation effect than the other elements. During oxidation, higher homogeneity resulted in the formation of a thin layer of Al complex oxide and a benefit to the oxidation resistance at the early stage, which was not observed in NbTi + CrTa. Consequently, Al exhibits superior oxidation resistance in NbTi-bearing RHEAs at lower addition amounts, as evidenced by the time exponent between NbTi + CrTa and NbTi + AlTa.

#### 4.2.3. The Elemental Effect of Mo and Ta during the Oxide Layer Formation

In the previous research with the CrNbTaTi system [25], the Ta addition from CrNbTi to CrNbTaTi made the oxide layers change from continuous Cr_2_O_3_ and TiO_2_ to repeating complex oxide layer structure with (Nb,Ta)_10_O_25_ and (Cr,Ta,Ti)_2_O_4_. The increased complexity of oxides might be induced by the similar formation energy of oxides. Ta also has a high oxygen solubility at 1000 °C [17,71]. In this work, Ta addition has a detrimental effect on the oxidation properties. In NbTi + AlMo, the outmost oxide layer was formed by an Al-related oxide (AlNbO_4_), which has the capacity to protect the underlying material [30,52,54]. Following the addition of Ta, the external oxidation region of NbTi + AlMoTa was composed solely of Ti-rich oxide and MoTiTa_8_O_25_. MoTiTa_8_O_25_ has been documented as a porous oxide with strong expansion from the alloy and induces the dispatch from the substrate [55]. Concurrently, the dilution of Al and the addition of Ta inhibited the formation of Al-related oxides.

Mo has relatively low oxygen solubility compared to other refractory elements [17]. During oxidation, Mo reacted with oxygen and formed MoO_3_. This oxide is volatile at over 800 °C [69]. This phenomenon will leave many porosities inside the oxide layer after evaporation, increase its surface area and make the oxygen penetrate the material quickly [21,38]. In NbTi + AlCrMo, an oxide layer can prevent oxygen from diffusing inward. In this context, the higher content of Mo in the dendrite region benefits the oxidation behavior. The low oxygen solubility results in the alloy absorbing less oxygen in the dendrite region [17,72]. In alloys with Mo, there is no Ti oxide precipitation in the internal oxidation layer. However, the oxygen reacted with Mo without the protective external oxide layer, forming volatile oxide. The continuous porosities formed at the original dendrite region provided an excellent environment to accelerate the oxidation, such as NbTi + AlMo and NbTi + AlMoTa.

### 4.3. Comparison to the RHEAs Reported Recently

**Figure 12:**
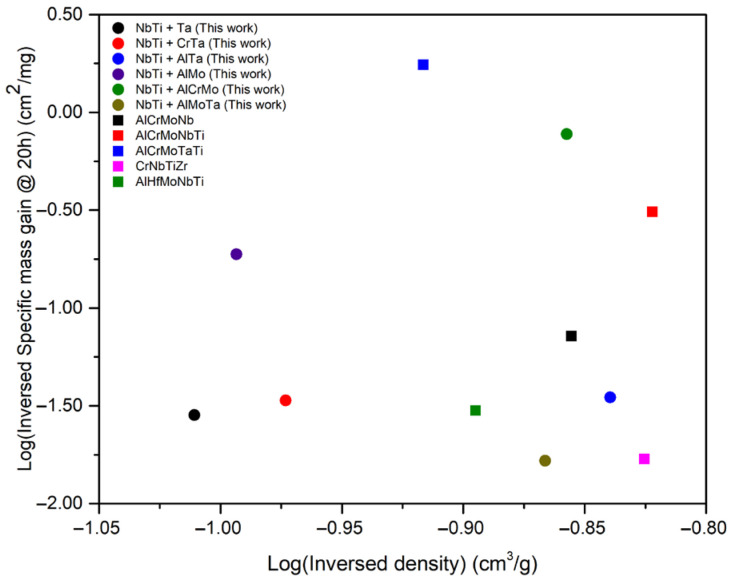
The comparison of the specific mass gain tested at 1000 °C for 20 h among NbTi-bearing RMEAs and RHEAs from this work, AlCrMo + Nb/NbTi/TaTi [25], CrNbTiZr [28] and AlHfMoNbTi [38] from the literature.

Figure 12 compares the oxidation performance of the NbTi-bearing RMEAs and RHEAs developed in this study with previously reported RHEAs tested under similar conditions (1000 °C for 20 h) [25,28,38].

The density is evaluated from the rule of mixture. Notably, NbTi + AlCrMo exhibits superior oxidation performance compared to compositions reported in the literature with similar elements (AlCrMoNbTi) [25]. This enhanced performance might be attributed to the formation of Laves phases at grain boundaries within the reported alloys. In those alloys, Al_2_O_3_ precipitates around the Laves phases, while CrN and TiN form along the grain boundaries. Conversely, the NbTi + AlCrMo developed in this study exhibits a fully single-phase microstructure, eliminating the aforementioned phenomenon.

While NbTi + AlCrMo demonstrates remarkable oxidation resistance, AlCrMoTaTi, reported elsewhere, surpasses its performance. This can be explained by the difference in the behavior of their respective oxides at elevated temperatures. Nb_2_O_5_ undergoes polymorphic transitions around 1000 °C, whereas Ta_2_O_5_ maintains a stable monoclinic structure up to 1350 °C [66,67]. The polymorphic transformations in Nb_2_O_5_ induce volume expansion, which can lead to crack formation within the oxide layer on NbTi + AlCrMo. However, the density of AlCrMoTaTi is much higher than NbTi + AlCrMo (7.2 and 8.25 g/cm^3^ for NbTi + AlCrMo and AlCrMoTaTi, respectively).

## 5. Conclusions

In the present study, six types of NbTi-bearing RMEAs and RHEAs were fabricated. The relationship between element effects, oxidation and segregation was discussed among these alloys. A higher content of Cr was beneficial due to the formation of continuous CrNbO_4_ and Cr_2_O_3_. Due to the segregation of Ti, TiO_2_ was mainly present in the interdendritic region. Mo and Ta segregated to the dendritic region, and Ta exhibited a detrimental effect on oxidation resistance in most cases. The dilution of the protective elements and segregation made the formation of a protective oxide layer during oxidation difficult. The effect of Mo addition on oxidation varied depending on the type of oxide layer present. In the presence of a protective oxide layer, low oxygen solubility could enhance oxidation resistance. However, in the absence of a protective oxide layer, the segregation of Mo could result in the formation of voids due to Mo-oxide evaporation. In this work, NbTi + AlCrMo showed good oxidation at a high temperature and low density. It can be a candidate for next-generation materials for high-temperature applications, such as aerospace and gas turbines.

## Figures and Tables

**Table 1:** The compositions of the fabricated alloy measured by EDS (in at%).

Alloy	Al	Cr	Mo	Nb	Ta	Ti
NbTi + Ta	-	-	-	33.1 ± 0.5	36.2 ± 0.3	30.7 ± 0.3
NbTi + CrTa	-	10.9 ± 0.5	-	27.1 ± 0.1	28.8 ± 0.2	33.2 ± 0.4
NbTi + AlMo	15.2 ± 0.4	-	26.9 ± 0.7	28.1 ± 0.2	-	29.8 ± 0.5
NbTi + AlTa	8.6 ± 0.4	-	-	30.1 ± 0.4	35.2 ± 1.2	26.1 ± 2.0
NbTi + AlCrMo	10.3 ± 0.3	21.0 ± 0.9	23.9 ± 1.0	23.4 ± 0.9	-	21.5 ± 0.7
NbTi + AlMoTa	5.1 ± 0.2	-	23.5 ± 0.9	25.0 ± 0.9	25.3 ± 0.9	21.1 ± 0.4

**Table 2:** Chemical compositions (in at %) of dendrite (D) and interdendritic (ID) region in six NbTi-bearing RMEA and RHEAs.

Alloy	Region	Al	Cr	Mo	Nb	Ta	Ti
NbTi + Ta	D	-	-	-	32.2 ± 0.4	37.8 ± 0.8	30.1 ± 0.4
	ID	-	-	-	32.2 ± 0.7	28.0 ± 0.3	39.8 ± 2.0
NbTi + CrTa	D	-	7.7 ± 1.4	-	29.8 ± 0.5	36.4 ± 1.2	26.2 ± 0.2
	ID	-	18.9 ± 0.1	-	20.3 ± 0.5	13.3 ± 0.6	47.4 ± 0.9
NbTi + AlMo	D	13.8 ± 0.7	-	31.0 ± 0.7	30.3 ± 0.3	-	24.8 ± 0.0
	ID	18.3 ± 0.2	-	23.9 ± 0.4	27.0 ± 0.1	-	30.8 ± 0.3
NbTi + AlTa	D	8.6 ± 0.0	-	-	31.3 ± 0.6	34.7 ± 0.3	25.5 ± 0.3
	ID	14.9 ± 0.7	-	-	27.7 ± 0.1	21.9 ± 1.6	35.5 ± 1.0
NbTi + AlCrMo	D	9.3 ± 0.1	16.7 ± 0.2	28.3 ± 0.2	24.6 ± 0.3	-	21.1 ± 0.4
	ID	15.3 ± 0.8	31.3 ± 0.4	8.8 ± 1.4	17.7 ± 0.7	-	26.9 ± 1.0
NbTi + AlMoTa	D	3.3 ± 0.1	-	25.0 ± 0.2	24.8 ± 0.1	29.2 ± 0.2	17.8 ± 0.1
	ID	12.2 ± 1.5	-	17.2 ± 1.2	22.7 ± 0.4	12.3 ± 2.0	35.6 ± 2.0

**Table 3:** The oxidation rate constant and time exponent of NbTi-bearing RMEAs and RHEAs.

Alloy Name	*k1*	*n1*	Period	*k2*	*n2*	Period
NbTi + Ta	5.62	0.55	1–9 h	2.45	0.89	9–20 h
NbTi + CrTa	5.88	0.46	1–14 h	1.05	1.12	14–20 h
NbTi + AlTa	5.79	0.34	1–7 h	2.19	0.84	7–20 h
NbTi + AlMo	1.27	0.57	1–4 h	1.77	0.32	4–20 h
NbTi + AlCrMo	0.33	0.34	1–7 h	0.21	0.59	7–20 h
NbTi + AlMoTa	9.12	0.76	1–15 h			

**Table 4:** Chemical compositions (in at %) of the indicated regions in Figure 5.

Region	Al	Cr	Mo	Nb	Ti	O
1	17.7 ± 1.0	9.8 ± 3.7	0.2 ± 0.1	2.8 ± 0.3	5.3 ± 1.0	64.2 ± 2.8
2	5.1 ± 0.3	25.0 ± 4.3	0.3 ± 0.2	2.4 ± 0.5	4.5 ± 1.7	62.7 ± 2.2
3	2.7 ± 0.2	3.3 ± 0.1	1.7 ± 0.1	17.8 ± 0.1	8.7 ± 0.3	66.0 ± 0.4
4	6.6 ± 0.4	17.0 ± 5.3	9.8 ± 1.7	12.3 ± 0.9	24.7 ± 3.3	(N)29.7 ± 3.0
5	6.8 ± 0.3	22.7 ± 1.2	9.0 ± 0.1	13.9 ± 0.3	29.7 ± 1.8	18.1 ± 0.1

**Table 5:** Chemical compositions (in at %) of the indicated regions in Figure 7.

Region	Al	Cr	Nb	Ta	Ti	O
1	-	-	2.1 ± 0.6	2.4 ± 0.7	30.7 ± 3.1	64.8 ± 3.9
2	-	-	10.5 ± 0.6	14.4 ± 0.6	8.5 ± 2.3	66.6 ± 1.7
3	-	-	4.0 ± 0.3	4.1 ± 0.4	38.6 ± 0.1	53.4 ± 0.7
4	-	1.6 ± 0.3	10.4 ± 0.4	13.6 ± 0.7	7.2 ± 0.9	63.2 ± 6.5
5	-	3.3 ± 0.4	7.7 ± 0.7	6.4 ± 0.4	19.3 ± 0.5	63.3 ± 0.3
6	-	3.2 ± 1.0	3.2 ± 0.4	4.7 ± 1.3	38.3 ± 0.1	50.8 ± 0.6
7	7.3 ± 0.3	-	4.1 ± 0.2	4.9 ± 0.2	15.0 ± 0.7	68.7 ± 1.4
8	0.2 ± 0.1	-	14.2 ± 0.9	12.5 ± 0.7	4.0 ± 0.2	69.0 ± 0.2
9	2.8 ± 0.5	-	9.5 ± 0.6	11.9 ± 0.5	9.8 ± 1.8	66.1 ± 1.4
10	5.3 ± 0.2	-	4.7 ± 0.9	5.8 ± 1.1	20.1 ± 2.2	64.1 ± 0.1
11	8.9 ± 0.3	-	3.3 ± 0.9	5.0 ± 0.2	23.7 ± 1.2	59.2 ± 0.8

**Table 6:** The fractions of oxides (in %) shown in Figure 8.

Alloy	Ta_2_O_5_	TiO_2_	AlTaO_4_	Al_2_TiO_5_	Nb_2_O_5_	Nb_4_O_5_	AlNbO_4_	TiNbO_4_	TiTaO_4_	CrNbO_4_
NbTi + Ta	41.8	57.6	-	-	0.6	-	-	-	-	-
NbTi + CrTa	29.52	41.02	-	-	0.04	-	-	-	-	29.42
NbTi + AlTa	7.9	9.8	10.1	2.7	7.6	1.4	38.7	11.6	10.3	-

**Table 7:** Chemical compositions (in at %) of the indicated regions in Figure 10.

Region	Al	Mo	Nb	Ta	Ti	O
1	13.6 ± 0.3	0.0 ± 0.0	13.1 ± 0.3	-	9.7 ± 0.5	63.7 ± 0.1
2	7.0 ± 0.3	0.4 ± 0.2	8.6 ± 0.6	-	19.2 ± 0.3	64.8 ± 0.7
3	1.3 ± 0.4	2.7 ± 0.4	24.9 ± 4.2	-	5.7 ± 1.1	65.4 ± 3.8
4	0.4 ± 0.1	4.0 ± 0.2	9.2 ± 0.3	12.1 ± 0.1	4.9 ± 0.1	69.3 ± 0.1
5	2.7 ± 0.7	1.9 ± 0.5	6.8 ± 1.1	8.1 ± 0.8	12.5 ± 2.3	68.0 ± 0.6

**Table 8:** The fractions of oxides (in %) shown in Figure 11.

Alloy	Ta_2_O_5_	TiO_2_	MoO_3_	AlTaO_4_	Al_2_TiO_5_	Nb_2_O_5_	AlNbO_4_	TiNbO_4_	TiTaO_4_
NbTi + AlMo	-	43	4	-	2.5	2.2	37.2	11.1	-
NbTi + AlMoTa	2.1	14.1	12.3	5.9	9.1	10.7	24.9	5.5	15.3

**Table 9:** The partition coefficient of each element in NbTi-bearing RMEAs and RHEAs.

Alloy	Al	Cr	Mo	Nb	Ta	Ti
NbTi + Ta	-	-	-	1.01	1.09	0.9
NbTi + CrTa	-	0.71	-	1.05	1.13	0.87
NbTi + AlMo	0.86	-	1.09	1.04	-	0.93
NbTi + AlTa	0.73	-	-	1.04	1.19	0.84
NbTi + AlCrMo	0.83	0.73	1.32	1.11	-	0.93
NbTi + AlMoTa	0.75	-	1.05	1.02	1.14	0.86

**Table 10:** The mixing enthalpies (kJ/mol) for the atomic pairs with each element in this work [63].

	Al	Cr	Mo	Nb	Ta	Ti
**Al**		−10	−5	−18	−19	−30
**Cr**	−10		0	−7	−7	−7
**Mo**	−5	0		−6	−5	−4
**Nb**	−18	−7	−6		0	2
**Ta**	−19	−7	−5	0		1
**Ti**	−30	−7	−4	2	1	

**Table 11:** The activity of alloying elements based on the composition in the dendritic (D) and interdendritic (ID) regions.

Alloy	Region	Al	Cr	Mo	Nb	Ta	Ti
NbTi + Ta	D	-	-	-	5.73 × 10^−4^ ± 8.53 × 10^−6^	3.06 × 10^−4^ ± 6.03 × 10^−6^	1.30 × 10^−3^ ± 1.01 × 10^−5^
	ID	-	-	-	6.03 × 10^−4^ ± 2.08 × 10^−6^	2.61 × 10^−4^ ± 8.70 × 10^−6^	1.45 × 10^−3^ ± 3.33 × 10^−5^
NbTi + CrTa	D	-	2.27 × 10^−3^ ± 5.86 × 10^−6^	-	5.31 × 10^−4^ ± 5.69 × 10^−6^	3.18 × 10^−4^ ± 5.46 × 10^−6^	1.14 × 10^−3^ ± 1.65 × 10^−5^
	ID	-	2.49 × 10^−3^ ± 1.21 × 10^−5^	-	5.56 × 10^−4^ ± 6.18 × 10^−6^	1.83 × 10^−4^ ± 5.38 × 10^−6^	1.54 × 10^−3^ ± 1.15 × 10^−5^
NbTi + AlMo	D	7.55 × 10^−6^ ± 1.01 × 10^−6^	-	6.06 × 10^−4^ ± 2.80 × 10^−5^	2.78 × 10^−4^ ± 6.79 × 10^−6^	-	2.24 × 10^−4^ ± 7.36 × 10^−6^
	ID	1.49 × 10^−5^ ± 4.64 × 10^−7^	-	3.48 × 10^−4^ ± 1.13 × 10^−5^	2.80 × 10^−4^ ± 3.21 × 10^−6^	-	2.65 × 10^−4^ ± 6.74 × 10^−6^
NbTi + AlTa	D	9.13 × 10^−7^ ± 4.14 × 10^−9^	-	-	6.28 × 10^−4^ ± 8.56 × 10^−6^	3.39 × 10^−4^ ± 3.67 × 10^−6^	6.85 × 10^−4^ ± 2.09 × 10^−6^
	ID	1.36 × 10^−6^ ± 9.96 × 10^−8^	-	-	6.68 × 10^−4^ ± 4.18 × 10^−6^	2.30 × 10^−4^ ± 1.77 × 10^−5^	7.98 × 10^−4^ ± 1.37 × 10^−5^
NbTi + AlCrMo	D	2.34 × 10^−6^ ± 6.77 × 10^−8^	4.14 × 10^−3^ ± 3.08 × 10^−5^	8.65 × 10^−4^ ± 1.44 × 10^−5^	1.91 × 10^−4^ ± 3.84 × 10^−6^	-	2.72 × 10^−4^ ± 7.10 × 10^−6^
	ID	3.50 × 10^−6^ ± 3.08 × 10^−7^	3.23 × 10^−3^ ± 1.72 × 10^−4^	3.62 × 10^−4^ ± 6.96 × 10^−5^	1.81 × 10^−4^ ± 8.17 × 10^−7^	-	5.71 × 10^−4^ ± 4.94 × 10^−5^
NbTi + AlMoTa	D	8.92 × 10^−7^ ± 1.11 × 10^−8^	-	2.28 × 10^−4^ ± 4.03 × 10^−6^	2.96 × 10^−4^ ± 2.99 × 10^−6^	1.97 × 10^−4^ ± 1.84 × 10^−6^	3.66 × 10^−4^ ± 2.41 × 10^−6^
	ID	2.63 × 10^−6^ ± 6.24 × 10^−7^	-	1.47 × 10^−4^ ± 1.44 × 10^−5^	3.37 × 10^−4^ ± 1.24 × 10^−7^	1.11 × 10^−4^ ± 1.52 × 10^−5^	5.41 × 10^−4^ ± 1.85 × 10^−5^

## Data Availability

Dataset available on request from the authors.
